# General dental practitioners' knowledge and opinions of snoring and sleep-related breathing disorders

**DOI:** 10.1038/s41415-021-3573-z

**Published:** 2021-11-12

**Authors:** Charlotte Leigh, Maurice Faigenblum, Peter Fine, Robert Blizard, Albert Leung

**Affiliations:** UCL Eastman Dental Institute, London, UK

## Abstract

**Aims** There is increasing recognition that sleep disturbances can affect lifestyle, economy and health. General dental practitioners (GDPs) can play a vital role in helping to identify at-risk patients through screening as well as aid in the management of these conditions. The aim of this study was to assess the knowledge of UK-based GDPs in relation to sleep-related breathing disorders (SRBDs).

**Material and methods** A questionnaire was developed to assess GDPs' knowledge of SRBDs and their current practice in relation to the management of SRBDs, and identify the factors associated with improved knowledge and management. A volunteer sample was recruited from social media platforms and conference attendees.

**Results** In total, 152 GDPs completed the questionnaire; 101 (66% [95% CI 59%,74%]) agreed that GDPs should ask their patients about sleep disorders, while only 82 (54% [95% CI 46%,62%]) confirmed that they actually asked their patients about the problem. On a 22-point knowledge scale, the mean score was 12.93 (95%CI 12.23, 13.63). Postgraduate education (F = 5.47; p = 0.001), increasing age (r = 0.23; p = 0.005) and GDP workplace (F = 4.98; p = 0.008) contributed to a higher knowledge score. GDPs were strongly in favour of having more information (N = 141; 93% [95% CI 87%,97%]).

**Conclusion** Although GDPs' knowledge of SRBDs was better than anticipated, they clearly felt a need for further information and training. There is a compelling case to include this subject in the undergraduate dental curriculum.

**Supplementary Information:**

Zusatzmaterial online: Zu diesem Beitrag sind unter 10.1038/s41415-021-3573-z für autorisierte Leser zusätzliche Dateien abrufbar.

## Key points

- Provides an overview of the role of GDPs in supporting patients with sleep-related breathing disorders (SRBDs).
- Highlights the need for GDPs to work closely with physicians in dealing with SRBDs.
- Presents evidence for the need of further education of GDPs in recognising and treating SRBDs.

**Supplementary Information:**

Zusatzmaterial online: Zu diesem Beitrag sind unter 10.1038/s41415-021-3573-z für autorisierte Leser zusätzliche Dateien abrufbar.

## Introduction

'Sleep-related disordered breathing is a common condition, with a wide spectrum of severity ranging from habitual snoring to obstructive sleep apnoea' (OSA).^1^ This is categorised 'by repetitive episodes of decreased or arrested respiratory airflow during sleep'.^2^ Snoring and OSA fall under this umbrella term, the latter of which can manifest as a potentially serious medical condition. Patients with sleep-related disordered breathing (SRDB) often complain of daytime sleepiness, interrupted sleep, snoring and breathing pauses.^3^

So-called 'simple snoring' has been defined as 'fluttering sound created by the vibrations of pharyngeal tissues or more generally a sound produced by the upper aero-digestive tract during sleep'.^4^ However, this definition is by no means universally accepted; there being less agreement in what is considered to be 'clinically relevant snoring'. The variations in study methodologies and the putative physiological and anatomical correlates within each snoring classification add to the discussion.^4^ An initial diagnosis of 'simple snoring' should alert the dental practitioner to investigate the possibility of SRBD. Disturbed sleep as a result of SRBD is characterised by a decrease in inspiration that lasts for at least ten seconds and can be partial (hypopnea) or total (apnoea). The severity of this condition is measured by the number of these events per hour; that is, the apnoea/hypopnea index (AHI). Patients who snore but have an AHI <5 tend to be classed as primary or habitual snorers.^4^ The increase in the number of apnoea/hypopnoea per hour can lead to severe interruption of breathing, known as OSA. Not all snorers will be diagnosed with OSA, but most of those diagnosed with OSA do snore. This has severe implications on the health of an individual.^5^ Snoring itself is not thought to pose a risk to health on its own, but as part of the continuum of an elevated risk of it developing into OSA, and this risk fact alone merits early investigation.^4^^,^^6^

Lechner *et al.* (2019) investigated a sample of non-institutionalised UK population between the ages of 18-100 and found that '30.4% of women and 38% of men self-reported that they snore at night; furthermore, 8.7% of men and 5.6% of women report that they have episodes when they stop breathing at night'. A small proportion (3.43%) of the study population did not know whether they experienced breathing pauses or snored during sleep. There was a significant increase in the rates of sleep apnoea and obesity in the UK over the last 20 years, concluding that 'sociodemographic and behavioural changes have likely contributed to this and sleep-related breathing is widely under diagnosed in the UK'.^7^

There is little consensus on specific diagnostic criteria related to snoring, which often relies on self-reported or partner-reported snoring rather than the stricter criteria related to OSA. Despite this long-standing awareness of snoring and the havoc it creates in many bedrooms, many believe snoring to be merely a social nuisance, without any adverse health consequences to the snorer.^8^

General dental practitioners (GDPs) can play three roles in managing SRBD: i) to act as gatekeepers by screening and supporting triage; ii) to make referrals onwards to medical colleagues; and iii) to be able to treat snoring and OSA with oral appliances.^9^^,^^10^^,^^11^ It is important to note that such treatment should be in coordination with the appropriate sleep physicians.

There are certain characteristics that increase the risk of snoring, including anything that increases the risk of upper airway infection or inflammation. Nasal obstruction, smoking or alcohol dependence, being male,^12^^,^^13^ an individual's body mass index (BMI; of at least 25 kg/m^2^)^14^^,^^15^ and neck circumference (17+ inches) are strongly correlated to SRBD.^4^ Factors can be assessed during a routine dental examination such as craniofacial morphology and size of the surrounding soft tissue structures (tongue, soft palate, lateral pharyngeal walls).^16^ The size of bony enclosures (mandible, maxilla and cervical spine) can affect the size of the pharyngeal lumen. Patients that suffer from hypoplasia and/or retro disc displacement of the mandible and maxilla have restricted space in the oropharyngeal cavity because the tongue, soft palate and soft tissues are displaced posteriorly.^17^ Together with the overall appearance and demeanour of the patient, including their level of obesity, degree of breathlessness and sleepiness during the day, these factors can be brought to their attention, stimulating a discussion and resulting in diagnosis and possible treatment.

A simple and widely used questionnaire, such as the Epworth Sleepiness Scale (ESS),^18^^,^^19^ can be completed either in the surgery or at home and can help to gauge the severity of SRBD both for the patient and GDP. An alternative questionnaire is the 'STOP-Bang' questionnaire. This can be used with patients suspected of suffering from OSA and can be used in the clinical setting. The scoring system takes into account age, BMI, neck circumference and gender.^20^ The score obtained from the questionnaires alerts the patient and the GDP to the effect of snoring and acts a marker to the success or otherwise of treatment. Consideration could be given to asking patients' sleeping partners, although most adult patients attend dental appointments alone.^15^

The primary role of GDPs is to maintain and improve oral health but also to be cognisant of the patient's general health. GDPs can screen patients deemed to be at risk of SRBDs and work with a recognised sleep physician in the provision of intraoral appliances when appropriate. Ramar *et al.* (2015) provided clinical practice guidelines of treatment on all forms of SRBD that are suitable with oral appliance therapy. Their recommendations included providing oral appliances (OAs) for adult patients who request treatment for snoring alone. They recommend that these appliances should be provided by a qualified GDP who has undergone appropriate training and that an OA should be supplied for OSA patients when continuous positive airway pressure is not tolerated.^21^^,^^22^ An OA is designed to introduce an advancement of the mandible, thus increasing oropharyngeal and hypo-pharyngeal dimensions, which encourages a smoother flow of air. A regular re-appraisal of the OA is needed to ensure compliance and comfort. Patients often report improvements in sleep quality and significant reductions in ESS are recorded in a high percentage of patients.^5^ Cooperation between general medical practitioners (GMPs) and GDPs is essential. The definitive diagnosis of SRBD needs to be undertaken by a suitably trained medical practitioner, who may then refer the patient onto/back to the GDP for any appropriate OA. The treatment of snoring or OSA should be done as a team approach and not by the GDP in isolation. A lack of knowledge and training regarding OSAs and cooperation between GMPs and GDPs results in a less effective response for patients. Correct diagnosis of simple snoring and OSA is therefore vital to ensure that patients receive appropriate treatment.^5^ Education for physicians and dentists alike on the impact of SRBDs will help its diagnosis and treatment.^23^ There would appear to be very little contemporary knowledge of SRBDs among GDPs in the UK; no undergraduate teaching currently occurs.^15^

The aim of this study was to investigate UK GDPs' knowledge regarding diagnosis and treatment of SRBDs.

## Methodology

A bespoke cross-sectional questionnaire was developed^24^ based upon previous studies.^25^^,^^26^ The questionnaire was designed to elicit information from GDPs in the UK about: i) demographics; ii) their knowledge of treating snoring and OSA; and iii) their current clinical practices. Potential respondents were assured of complete confidentiality and anonymity. Approval was applied and granted through the University College London (UCL) Low Level Ethics Committee (UCL NO: 6552/005).

Questions for the survey were distributed under five major headings:

1. Knowledge
2. Opinions
3. Education
4. Health professional and clinical practice
5. Demographic information.

Respondents were asked about their knowledge of current guidelines for SRBDs, clinical practice and educational experiences, all of which can be classified under the heading of 'sleep dentistry'.^27^

Poor knowledge was defined as scoring <20% of the total knowledge questions in the SRBD questionnaire. This figure was based on the proposition that all participants would have a basic dental knowledge and participants may have an awareness of SRBDs from the media^28^ and/or family members. A five-point Likert scale was used for some questions, where the opinion of respondents was sought.^29^

### Sample size calculation

Due to the paucity of previous studies, the sampling parameters for this study were designed around the calculation of a suitable sample size and the predicted confidence interval (CI).

There are approximately 41,000 GDPs in the UK;^30^ we estimated that 90% of GDPs will not ask about their patients' sleep habits. The same proportion will have a poor knowledge of SRBDs. In order to estimate this 90% with a precision of ± 5%, a sample of 139 GDPs was required.^31^

### Sample recruitment

An opportunist sample was recruited from both electronic sources (Facebook) via Survey Monkey^32^ and hard copy invitation at conferences and CPD events. Due to the variety of methods of distribution of the questionnaire, it was not possible to either predict or report the response rate. The collection of data through online surveys has limitations due to the unknown number of potential respondents who receive the link to the questionnaire

### Data analysis

Descriptive data were summarised with mean (SD) and N (%) as appropriate. The consistency of the questionnaire was examined by Cronbach's alpha test.^33^ Group comparisons were carried out with analysis of variance (ANOVA) and X^2^ test, and relationship between continuous variables by Pearson's correlation coefficient using SPSS software version 25 (IBM Corp. Released 2017. IBM SPSS Statistics for Windows, Version 25.0. Armonk, NY: IBM Corp). The analysis of the paper and electronic questionnaires was carried out in the same way. Qualitative data were analysed thematically.

## Results

The survey generated 154 responses which comprised 109 questionnaires completed on Survey Monkey and 45 questionnaires completed on paper, of which two were discarded (respondents were specialists and not GDPs). There was no significant demographic difference between the two groups defined by their method of recruitment, except that the paper group tended to be proportionately slightly over-represented in mixed practices (Table 1).

**Table 1:** Sample demography

Sample size (N = 152)	Mean (SD) or N(%)
Age	39.8 (12.67)
Gender	Male: 68 (45%)
Country of dental school	UK: 116 (78%)
Working environment	NHS: 24 (16%) Mixed practice: 92 (60%) Private practice: 36 (24%)
Source of information	Electronic: 109 (72%) Paper: 43 (28%)

### Internal consistency of the knowledge scale

**Fig. 1:**
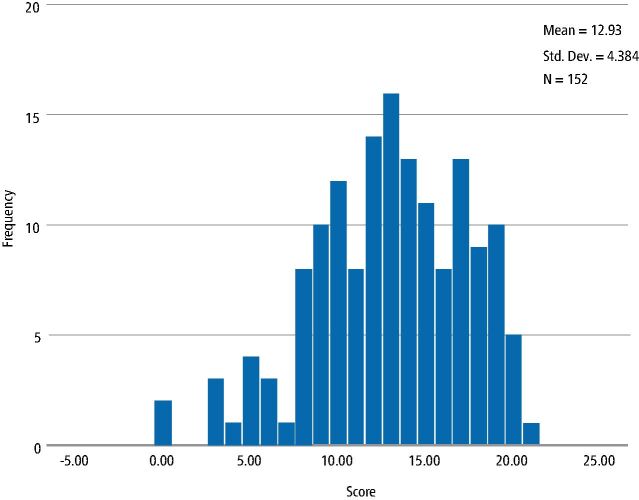
Distribution of the SRBD knowledge score from all respondents

Cronbach's alpha was calculated for the 22 individual items. This was found to be 0.81. According to George and Mallery (2003),^34^ 'this value indicated that the scale had good internal consistency to be used for group comparisons'. Each questionnaire completed was given a final score on the correct answers for the knowledge questions. Figure 1 shows the distribution of the correct scores achieved.

From Table 2, it can be seen that the following variables are associated with a higher knowledge score: place of work has a significant effect on the knowledge score; increasing age was associated with a higher score; and knowledge among GDPs working in private practice was the highest. GDPs who had heard of sleep dentistry scored higher. GDPs who had attended a postgraduate course scored higher than those that had not.

**Table 2:** Potential associations with the SRBD knowledge

Sample size = 152		Mean (SD)/N(%)	Test statistic	P value
Age	Years	-	R = 0.23	0.005
Sex	Male	12.83 (4.37)	F = 0.05	0.82
	Female	13.00 (4.43)		
Origin	UK	12.87 (4.28)	F = 0.05	0.94
	Non-UK	12.80 (5.01)		
Workplace	NHS	11.38	F = 4.98	0.008
	Mixed	12.63		
	Private	14.72		
Heard of SRBD	Yes	14.6 (4.51)	F = 14.387	<0.001
	No	11.93 (4.00)		
Postgraduate course	Yes	15.15 (4.22)	F = 5.47	<0.001
	No	12.13 (4.18)		

**Fig. 2:**
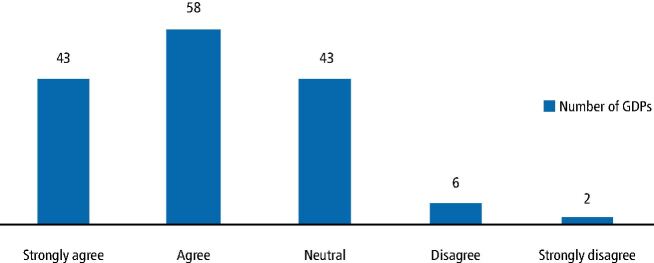
Respondents believe that GDPs should ask patients about their sleep habits

Figure 2 illustrates that the majority of respondents (n = 101 [66% (95% CI 59%, 73%]) agreed, based on a scale (where 1 = strongly agree and 5 = strongly disagree),^33^ that GDPs should ask patients about their sleep habits. GDPs believe it is important and thus within their remit to ask patients about their sleep habits.

**Fig. 3:**
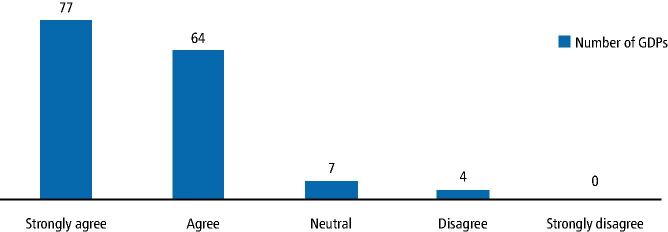
Respondents' replies to action taken by them if a patient mentioned they suffered from a SRBD

Minimal qualitative data were collected throughout the questionnaire. In response to a question about what action respondents would take if presented with a patient who mentioned they suffered from SRBD, the vast majority reported they would refer the patient for specialist opinion (n = 115; 76% [95% CI 68%, 82%]). These minimal qualitative data are represented in Figure 3 and examples of these comments are included. A thorough analysis of this data was not necessary due to the limited data collected:

- 'Refer to GMP for specialist investigations (sleep study) if appropriate'
- 'Initially refer but make an appliance if suitable'
- 'Provide brochures for snoring assessment and if patient is interested a second visit will be made for stages and financial aspect to make a snoring device'.

**Fig. 4:**
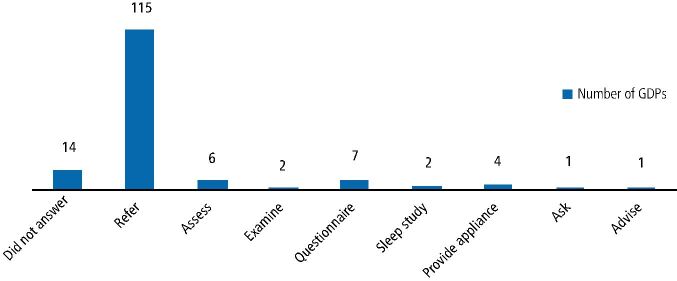
Respondents believe more information about OSA and snoring should be provided to GDPs

Figure 4 illustrates that the vast majority of respondents (n = 141; 93% [95% CI 88%, 96%]). Unsurprisingly, GDPs who had heard of sleep dentistry (n = 57; 37.5%) scored higher than those who were uninformed and GDPs who had attended postgraduate courses (n = 31; 20.4%) scored higher than those who had not. GDPs agreed or strongly agreed (n = 141; 93%) that more professional information should be available.

The results show that the knowledge of GDPs is better than anticipated and surprisingly more GDPs were discussing snoring and OSA with their patients than was expected.

## Discussion

### Knowledge

The 22 knowledge questions created for the questionnaire mainly focused on snoring, as this is more common and has a greater potential to be treated by a GDP. It was interesting to observe that many knowledge questions had been answered correctly. This would indicate that GDPs have some theoretical knowledge about snoring but this may come from a variety of non-evidence-based sources including the patient, their family members and the media. The theoretical knowledge exhibited by the respondents may be because of previous interest in this topic and attendance at CPD courses, experience of working alongside sleep physicians or having clinical experience of treating/monitoring patients with SRBDs; this is consistent with the positive relationship between knowledge and age.

Nearly all GDPs were aware of the impact that lifestyle choices can make on SRBDs; 93.4% correctly answered that weight loss is advised in the treatment of snoring and OSA. When asked whether patients who snore but have an AHI less than five are classed as primary or habitual snorers, GDPs were unaware of this, indicating a lack of technical knowledge; they were unaware of this simple breathing scale (AHI) and significance of the figure.

It was unsurprising to find that only a small number of respondents had any knowledge of 'sleep dentistry',^28^ which can be defined as the role a GDP, with appropriate training, can undertake to support both patients suffering from SRBDs and sleep physicians treating these patients. Similarly, it was predictable that only a small number of respondents had attended postgraduate courses to learn more about SRBDs as the topic is rarely perceived to be important when deciding on future postgraduate dental education. There is very little literature available, particularly in the UK dental press, regarding SRBDs and this subject is unlikely to be spoken about at dental conferences.^35^^,^^36^

### Opinions (education)

Undergraduate teaching of sleep, upper airway disorders and management of sleep disorders is notoriously poor and largely insufficient.^37^ Bian (2004)^25^ and Ivanhoe *et al.* (2003)^38^ reported only 31% of undergraduates have been taught about it in the USA. The results of this current study concur with this. Most respondents (n = 139; 91.4%) believe that dental schools should teach more about OSA. Current literature reveals no studies dedicated to investigating the teaching of sleep dentistry in the UK. The management of SRBDs is not detailed in 'The first five years' published by the General Dental Council (GDC) nor consistently part of the American undergraduate school curriculum. Lack of teaching at an undergraduate level may be a worldwide issue. When questioned, ninety-five (62.5%) GDPs had not heard of sleep dentistry; not only is the undergraduate education lacking in this regard, but also this subject is not widely discussed in the dental media. This study shows SRBDs are relevant to a GDP and there is a desire among practitioners to learn more; as a newly evolving area of dentistry, it should be incorporated into the dental undergraduate education. When asked, a majority of GDPs (n = 139; 92%) strongly agreed or agreed that dental schools should be teaching more about OSA, with only one dentist strongly disagreeing. A majority of GDPs (n = 141; 93%) also strongly agreed or agreed that more information should be provided to help GDPs, with only four dentists disagreeing.

### Health professional and clinical practice

See online supplementary information. GDPs without relevant clinical information about snoring may be unable to identify key features of this condition and thus play a role in its management. When questioned, 101 (66%) GDPs either agreed or strongly agreed that they should be asking about the sleep habits of their patients. However, it is most likely that patients do not perceive the management of snoring and OSA as being within the scope of practice of the dentist. Many GDPs (n = 79; 52%) were unsure about the efficacy of screening methods such as the use of the ESS. These results suggest that GDPs are interested in knowing more about the management of SRBDs and view it as important, but are unsure of the role they should/could play and the use of screening tools they can use during routine examinations (clinical features and questionnaires). As a health professional, the GDP should be adopting a more holistic approach to screening which will inevitably include the signs and symptoms of SRBDs.

### Role of GDP

#### Screening

A small majority of GDP respondents (n = 79; 51.3%) believed that GDPs should be screening patients using methods such as the ESS and 101 (65.6%) agreed that GDPs should ask patients about their sleep habits.

In the authors' opinion, SRBD is perceived as an embarrassing condition and patients may be reluctant to attend their GMP to seek advice for fear of wasting the GMP's time. The inclusion of sleep inadequacy, sleep disruption, health problems, ongoing psychological issues and/or work environment contribute to the evidence.^39^ Dentists are arguably one of the most commonly visited health professionals and are ideally placed to question patients about medical and lifestyle conditions (and possibly family members) and review the patient regularly.

#### Referral

When faced with a patient who complained of a sleep breathing disorder, 76% (n = 116) of GDPs said they would refer most commonly to the GMP (Fig. 3). It is important to recognise that a definitive diagnosis of OSA can only be made by a medical practitioner. The use of overnight polysomnography or other sleep assessments prescribed by physicians are necessary and imperative to a positive OSA diagnosis. Many studies have advocated that managing OSA is a team approach and dentists should work closely with physicians.^10^^,^^40^^,^^41^^,^^42^ Although only one study in the literature^26^ examined cooperation between dentists and physicians, it should be pointed out that referral is not uni-directional. Medical assessment of snorers with an overnight sleep study or specialist referral is often unavailable due to financial constraints and lack of available facilities. If an estimated 25-35% of the adult population snore and 30-35% of snorers suffer from OSA, busy sleep units and overburdened NHS teams would be unlikely to welcome a swathe of referrals just so GDPs can treat simple snorers.^5^ Physicians should be made aware of GDPs in their locality who are able to construct suitable devices and assist in the management of these patients. GDPs can play a crucial role in assisting in the diagnosis by identifying risk factors with their expertise in the knowledge of craniofacial structure. This is both beneficial to the patients' general health, reduces the burden on the overworked medical system and ultimately ensures the best care for the patient.

#### Advice

Lifestyle modification including reducing alcohol, smoking and reducing weight have been shown to have a positive outcome on the severity of OSA and snoring. When taking a more holistic approach, the GDP is in the ideal position to discuss this with the patient and make the appropriate referral if needed. This precedent has been previously used in connection with smoking cessation.^43^

#### Mandibular advancement devices

Mandibular advancement (MA) devices provide opportunities for dentists to actively participate in SRBD care. It was interesting to note that only four (2.6%) respondents reported that making an OA for a patient with a diagnosed SRBD would be appropriate. Most patients who snore and use an MA report a reduction in excessive daytime sleepiness and improvements in sleep quality.^44^ This suggests that through further education, even more GDPs will understand the importance of the role of the GDP in managing SRBDs for patients^11^^,^^45^ and how they can do this in general practice. The qualified dentist should be encouraged to continue their education in sleep dentistry and seek appropriate accreditation.^23^

#### Duty of care

A review of the literature demonstrated disparity between the way SRDB is viewed in the USA and the UK.^21^^,^^22^^,^^26^ The setting up of specialist societies, which have established an acknowledged pathway for the diagnosis and treatment of SRDBs, should be encouraged. This constitutes an implied duty of care regarding SRBDs, which is specifically absent from GDC regulations. Dental professionals 'have not been given any guidance on how to differentiate between patients who may be treated without further reference to medical colleagues (ie simple snorers), and those who should be referred for specialist assessment'.^5^ Asking patients about their sleep patterns might be unexpected and regarded by patients as an intrusion into their personal lives. However, there are precedents for this; for example, investigation into smoking habits^46^ and alcohol intake.^47^ The GDP needs to balance the undiagnosed condition of 'simple snoring' with the potential to over-medicalise it. In the UK, indemnity organisations allow GDPs to provide snoring appliances after additional training at no additional cost. Therefore, acknowledging the fabrication of devices is within the remit of a GDP. Each individual GDP should check with their indemnity organisation to ensure the appropriate cover is available before offering this service.

### Study limitations

This study only focused on GDPs based in the UK. As the GDC numbers of respondents were not checked against their legitimate participation, some specialists or retired GDPs may have completed the questionnaire.

The lack of data currently available, particularly reflective of the UK dental community, needs to be increased to enable more predictable parameters for calculating the likely outcomes for future studies.

Respondents completed the questionnaires online and in person, thus making a response rate impossible to calculate. Despite explicit instructions to complete all the questions, some participants did not answer all the questions or complete the demographic information. Their responses were included in the data set.

## Conclusion

This study on SRBDs is believed to be the first of its kind in the UK. It reveals a lack of knowledge among GDPs who are at the frontline treating patients on a regular basis. More educational courses should be available for GDPs to encourage them to incorporate screening into their daily practice. Despite its limitations, this research provides an interesting insight looking at the role GDPs can play within the wider health community to help support and treat these patients appropriately. If SRDB is to be taken seriously, it behoves the authorities to include this subject in the undergraduate dental curriculum and to work cooperatively with the UK-based sleep societies to raise awareness and standards.

## Supplementary Information

Final Questionnaire (PDF 467KB)
