# Supplementary material for: General dental practitioners' knowledge and opinions of snoring and sleep-related breathing disorders
Source: Br Dent J. 2021 Nov 12;231(9):569–74. doi: 10.1038/s41415-021-3573-z (PMC8589666; doi:10.1038/s41415-021-3573-z)
Supplement: Supplementary file 1 — Final Questionnaire (PDF 467KB) [file 41415_2021_3573_MOESM1_ESM.pdf]

Final Questionnaire

Questionnaire assessing GDPs knowledge of Snoring and Obstructive Sleep Apnoea

Title of Study:
An Investigation of the knowledge of Snoring and Obstructive Sleep Apnoea affecting patients amongst GDPs by means of a questionnaire – MSc Study
Department: Eastman Dental Institute
Name and Contact Details of the Researcher(s):Dr Charlotte Leigh (charlotte.leigh.15@ucl.ac.uk)

My name is Charlotte Leigh and I am undertaking this research project as part of my Master's degree in Restorative Dental Practice at UCL Eastman Dental Institute. You have been invited to take part in this research project about Snoring and Obstructive Sleep Apnoea (OSA) as a general dental practitioner (GDP). Please ensure you read the following information carefully and ask for any clarification necessary. Please take time to ensure you are happy to participate.

The purpose of the study is to ascertain the knowledge of **GDPs** with regards to snoring and OSA. This includes whether GDPs are aware of the methods to treat Snoring and OSA and how to access those methods. With the data collected in this study, I aim to develop a series of questions that GDPs can ask their patients in order to evaluate/identify those patients at high risk of snoring and OSA.

You have been chosen to volunteer for this study because you are a **GDP practicing in the UK**. All data will be collected anonymously and no personal information will be recorded. It is up to you whether you decide to take part, this survey is entirely voluntary. Participants have the right to withdraw from the study at any time without providing a reason. This questionnaire will take approximately 10 minutes to complete and please answer all the questions.
The study is broken down into knowledge based questions and please select one statement (true/ false) and opinion based questions (agree/ disagree).
This survey is exploring a new area of research and by taking part GDPs will have the opportunity to shape new guidelines to help them and their colleagues understand sleep disturbances.

If you wish to raise a complaint please contact Charlotte Leigh. If you feel your complaint has not been handled please contact Dr Peter Fine

All your responses to the questionnaire will be kept strictly confidential in a password protected computer. You will not be able to be identified in any ensuing reports or publications. Confidentiality will be respected subject to legal constraints and professional guidelines.

Results will be collated and a report prepared by December 2019. If you would like to be informed of the results of the study please leave your email address at the end of the questionnaire in the space provided.
Data collected during this project might be used for additional or subsequent research.
If you require any further information please contact charlotte.leigh.15@ucl.ac.uk

Thank you for reading this information sheet and for considering taking part in this research study.

1. What is your age?
……………………………………..

2. What is your gender? Please tick one box only

- ☐ Male
- ☐ Female

- ☐ Would rather not say

3. Where did you complete your undergraduate dental training?

School ………………………………..

Country ………………………………

4. Which region of the UK do you practice? Please tick one box only

- ☐ North East
- ☐ North West
- ☐ London
- ☐ Midlands
- ☐ South East
- ☐ South West
- ☐ Scotland
- ☐ Wales
- ☐ Northern Ireland

5. Describe your working environment: (Please tick the single most appropriate box)
- ☐ NHS
- ☐ Mixed Practice
- ☐ Private Practice

6. The most consistent risk factor for snoring is being male
- ☐ True
- ☐ False
- ☐ Don't know

7. Snoring is not considered a precursor to the development of obstructive sleep apnoea (OSA)
- ☐ True
- ☐ False
- ☐ Don't know

8. There is an association between snoring frequency and cardiovascular risk factors
- ☐ True
- ☐ False
- ☐ Don't know

9. Snoring intensity does not predict the likelihood of falling asleep at the wheel and traffic accidents.
- ☐ True
- ☐ False
- ☐ Don't know

10. Treating snoring early on does not prevent the development of more serious breathing related difficulties.
- ☐ True
- ☐ False
- ☐ Don't know

11. Snoring is a common worldwide with a prevalence rates ranging from 2% to 85% depending on diagnosis age gender and population

- ☐ True
- ☐ False
- ☐ Don’t know

12. Obstructive Sleep Apnoea (OSA) is defined as the temporary cessation in nasal and /or oral airflow coinciding with cessation of respiratory effort

- ☐ True
- ☐ False
- ☐ Don’t know

13. Hyponoea is know as partial collapse of the upper airway for 10 seconds or more during sleep is known as

- ☐ True
- ☐ False
- ☐ Don’t know

14. The severity of OSA is measured by the number of these events per hr known as the apnoea /hypopnoea index (AHI)

- ☐ True
- ☐ False
- ☐ Don’t know

15. Patients who snore but have an AHI less than five are classed as primary or habitual snorers

- ☐ True
- ☐ False
- ☐ Don’t know

16. Weight reduction is often recommended in the treatment of primary snoring and OSA

- ☐ True
- ☐ False
- ☐ Don’t know

17. The estimated prevalence of OSA in adults is between 2 - 10%

- ☐ True
- ☐ False
- ☐ Don’t know

18. Enlarged soft palate and neck diameter do not increase the likelihood of OSA

- ☐ True
- ☐ False
- ☐ Don’t know

19. Alcohol consumption may worsen OSA

- ☐ True
- ☐ False
- ☐ Don’t know

20. OSA is not associated with hypertension

- ☐ True
- ☐ False
- ☐ Don’t know

21. Patients with OSA have to declare this to the DVLA and have a higher incidence of motor accidents
- ☐ True
- ☐ False
- ☐ Don’t know

22. Sleep study is the gold standard in diagnosing OSA
- ☐ True
- ☐ False
- ☐ Don’t know

23. OSA can be diagnosed by a GDP
- ☐ True
- ☐ False
- ☐ Don’t know

24. Continuous positive airway pressure (CPAP) is the gold standard for treating obstructive sleep apnoea

- ☐ True
- ☐ False
- ☐ Don’t know

25. Many patients with severe OSA have no daytime sleepiness and actually claim to have good and restful sleep
- ☐ True
- ☐ False
- ☐ Don’t know

26. Oral Appliances are a modality of treatment for snorers and OSA patients
- ☐ True
- ☐ False
- ☐ Don’t know

27. Appliances can be made by a GDP for patients suffering from snoring / OSA
- ☐ True
- ☐ False
- ☐ Don’t know

28. Dentists should ask patients about their sleep habits
- ☐ Strongly Agree
- ☐ Agree
- ☐ Neutral
- ☐ Disagree
- ☐ Strongly Disagree

29. Dentists should be screening patients using methods such as the Epworth sleepiness scale
- ☐ Yes
- ☐ No
- ☐ Don’t know

30.Dental Schools should teach more about OSA
- ☐ Strongly Agree
- ☐ Agree
- ☐ Neutral

- ☐ Disagree
- ☐ Strongly Disagree

31. More information / courses should be provided to help GDPs in this field
    - ☐ Strongly Agree
    - ☐ Agree
    - ☐ Neutral
    - ☐ Disagree
    - ☐ Strongly Disagree

32. Had you heard of Sleep Dentistry
    - ☐ Yes
    - ☐ No

33. Have you ever discussed snoring/ OSA with your patients?
    - ☐ Yes
    - ☐ No

34. If a patient mentioned they suffered from a sleep breathing disorder what would you do?

35. Have you attended any post graduate courses?
    - ☐ Yes
    - ☐ No

Which course?

Thank you very much for completing this questionnaire based survey. I can assure you that all data will be stored anonymously. If you would like to hear about the results of this study please leave your email address below.

Charlotte Leigh
